# Unifying p*K*_a_ and Protonation Prediction with Sequence-Based Deep Learning

**DOI:** 10.1021/acs.jcim.6c00556

**Published:** 2026-05-29

**Authors:** Charlotte Infante, Jieyu Lu, Xiaolin Pan, Song Xia, Yingkai Zhang

**Affiliations:** † Department of Chemistry, 5894 New York University, New York, New York 10003, United States; ‡ Simons Center for Computational Physical Chemistry at New York University, New York, New York 10003, United States; § NYU-ECNU Center for Computational Chemistry at NYU Shanghai, Shanghai 200062, China

## Abstract

Predictions of p*K*_a_ values provide insights into key aspects of molecular behavior, including solubility, lipophilicity, and binding affinity. Despite their importance, experimental microscopic p*K*_a_ data remain scarce, creating a bottleneck in the training of accurate prediction models. In addition, inconsistent terminology across commonly used data sets hinders effective model development and benchmarking. While recent advances have been driven largely by graph-based neural networks, the potential of sequence-based deep learning for p*K*_a_ prediction remains underexplored. T5Chem, a sequence-based multitask chemical reaction model, offers an attractive way to cast molecular protonation/deprotonation as a language modeling task and to couple microstate generation with subsequent p*K*_a_ estimation. To pursue this direction, we introduce pKaCHU (p*K*_a_ data that are combined, honed, and updated), a curated data set comprising 9000 experimentally derived microscopic p*K*_a_ entries with ionization-state annotations. We also present T5pKa, a text-based transformer model for small-molecule p*K*_a_ prediction built on T5Chem. T5pKa leverages multitask learning to enumerate microstates, enabling both protonation and deprotonation to be predicted by a single sequence-to-sequence model, and then predicts microscopic p*K*_a_ values from the resulting microstate pairs using a separate regression model. Across benchmark data sets, T5pKa achieves performance comparable to established p*K*_a_ prediction tools and published models while offering the advantage of a unified multitasking framework for microstate enumeration and microscopic p*K*_a_ prediction.

## Graphical abstract

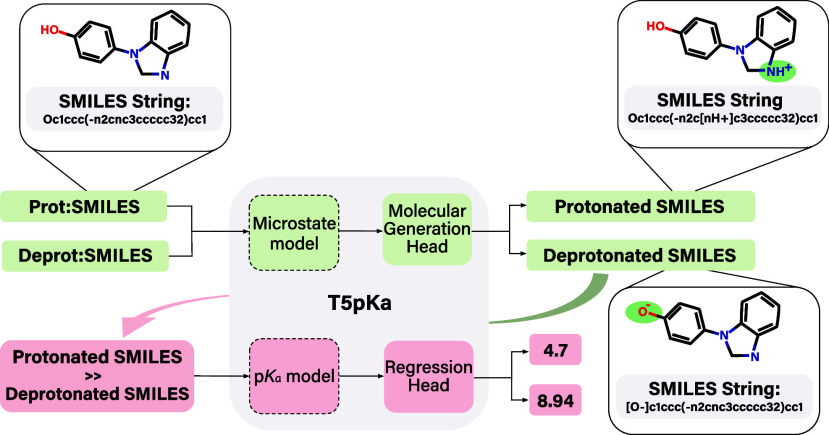

## Introduction

The dissociation of a proton from a molecule, quantified by the negative logarithm of the acid dissociation constant (p*K*_a_), plays a pivotal role in elucidating molecular behavior, guiding drug design, and optimizing chemical reactions.^1–3^ Accordingly, p*K*_a_ values are measured experimentally using techniques such as potentiometric titration, electrophoresis, and nuclear magnetic resonance (NMR),^4^ while computational methods are increasingly used to rapidly predict the p*K*_a_ of compounds in the virtual compound libraries prior to their synthesis^5^ to accelerate the drug discovery pipelines. Nevertheless, p*K*_a_ remains a challenging property to predict due to factors like conformational flexibility, structural symmetry, tautomerism, and neighboring titratable groups influencing the p*K*_a_ of an ionizable group.^6–9^ Because experimental methods vary in their ability to delineate the precise site of ionization, it is useful to distinguish between macroscopic p*K*_a_, which reflects the overall ionization behavior of a compound, and microscopic p*K*_a_, which refers to the equilibrium between specific protonation microstates at defined ionizable sites.^7,10^

Despite the broad importance of p*K*_a_, inconsistencies in terminology and incomplete ionization annotations have led to substantial ambiguity in existing data sets. For example, distinctions such as “acidic” versus “basic” p*K*_a_ infiltrated data sets without standardized definitions or explicit site-specific ionization assignments, hindering their integration and interpretation. Similarly, the sensitivity of p*K*_a_ values to differences in solvent systems, temperature, and extraction methodologies underscores the necessity of precise documentation and systematic organization within p*K*_a_ databases.^11–15^ Consequently, accurate p*K*_a_ prediction must be treated as a joint problem involving both ionization-site identification and numerical p*K*_a_ estimation under well-defined experimental conditions.

To address the inconsistencies present in existing p*K*_a_ data sets, we curated pKaCHU (p*K*_a_ data that are combined, honed, and updated) containing 9000 experimentally derived microscopic p*K*_a_ entries measured under aqueous conditions. This data set is designed to eliminate ambiguous terminology and explicitly represent site-specific ionization. In particular, pKaCHU represents sequential (de)­protonation of polyprotic molecules based on each p*K*_a_ value by encoding each microstate using simplified molecular input line entry system (SMILES)^16^ notation. By integrating and reconciling data from multiple literature sources, leveraging structure and charge information, and enforcing consistent experimental conditions whenever possible, pKaCHU provides a more chemically consistent foundation for microscopic p*K*_a_ prediction.

Computational p*K*_a_ prediction involves physics-based and empirical strategies, each offering distinct advantages. QM methods can provide physics-based estimates, but their computational cost limits throughput, even in hybrid frameworks such as Qupkake that couple QM calculations with graph neural networks.^17^ In contrast, empirical approaches are substantially faster and have achieved strong predictive performance. These encapsulate linear free energy relationship (LFER)-based commercial tools such as Epik^18^ and ACD/Laboratories,^19^ as well as quantitative structure–activity relationship (QSAR)-style models built from molecular descriptors, fingerprints, or graph representations.^20,21^ For example, Baltruschat and Czodrowski^22^ developed an open-source random-forest model for macroscopic p*K*_a_ prediction in monoprotic molecules. More recently, graph-centered methods such as MolGpKa,^23^ Graph-p*K*_a_,^8^ pKaSolver,^24^ MF-Sup-p*K*_a_,^25^ GR-p*K*_a_,^26^ and Uni-p*K*_a_ ^27^ have further advanced this area, while related efforts have also expanded p*K*_a_ modeling to supra-p*K*_a_ estimation,^28^ curated or noise-reduced data sets,^29^ and nonaqueous solvent prediction.^30^ Despite these advances, most existing methods still rely on predefined ionization rules,^18,19^ substructure-matching schemes,^23,27^ external ionization-state enumeration tools,^24^ or indirect free-energy formulations.^27^ While these choices can improve chemical plausibility and efficiency, they may also require continued manual curation or depend on fixed ionizable-group inventories. In addition, some existing approaches depend primarily on training pipelines built from calculated^23^ rather than carefully standardized experimental p*K*_a_ labels. While such strategies can provide large training sets, they may also limit the model’s ability to learn directly from experimental results. Collectively, these approaches do not fully exploit broader chemical relationships, particularly protonation-state changes as reaction-like transformations between molecular microstates. A sequence-based modeling framework that can learn such relationships directly from data could better capture chemically meaningful relationships between molecular microstates and also enable the flexible construction of sequential protonation and deprotonation pathways. Yet, such sequence-based approaches remain largely unexplored for p*K*_a_ prediction.

Motivated by the success of transformer-based models^31^ in molecular representation learning,^32–34^ we explore sequence-based modeling for ionization-aware prediction. Specifically, we present T5pKa, a text-based transformer model^35^ for small-molecule p*K*_a_ prediction built on the T5Chem^36^ architecture. We also leverage transfer learning from pretrained chemical language models, enabling T5pKa to exploit large-scale molecular knowledge while adapting to p*K*_a_-specific tasks. Unlike prior approaches that rely on predefined ionization rules or substructure matching schemes, T5pKa learns ionization behavior directly from data within a shared text-based framework. The framework integrates (i) microstate enumeration via sequence-to-sequence prediction and (ii) microscopic p*K*_a_ estimation via transformer-based regression on microstate pairs. The sequence-to-sequence model is trained to perform both protonation and deprotonation through task prefixes, enabling iterative application to construct sequential ionization pathways for polyprotic molecules (Figure 1). In contrast to approaches that model p*K*_a_ indirectly through free energy relationships, T5pKa directly predicts p*K*_a_ values from learned representations of ionization states. Together, these features make T5pKa a flexible alternative to existing p*K*_a_ prediction methods by providing directly learned microstates, leveraging transfer learning from broader chemical tasks, and reducing reliance on manually specified ionization rules. Further, we evaluate T5pKa on established benchmarks and compare it against widely used commercial tools and published machine-learning models. Finally, we perform model interpretation analysis to highlight chemical features associated with predicted microscopic p*K*_a_ values and to characterize model behavior across diverse functional groups.

**Figure 1:**
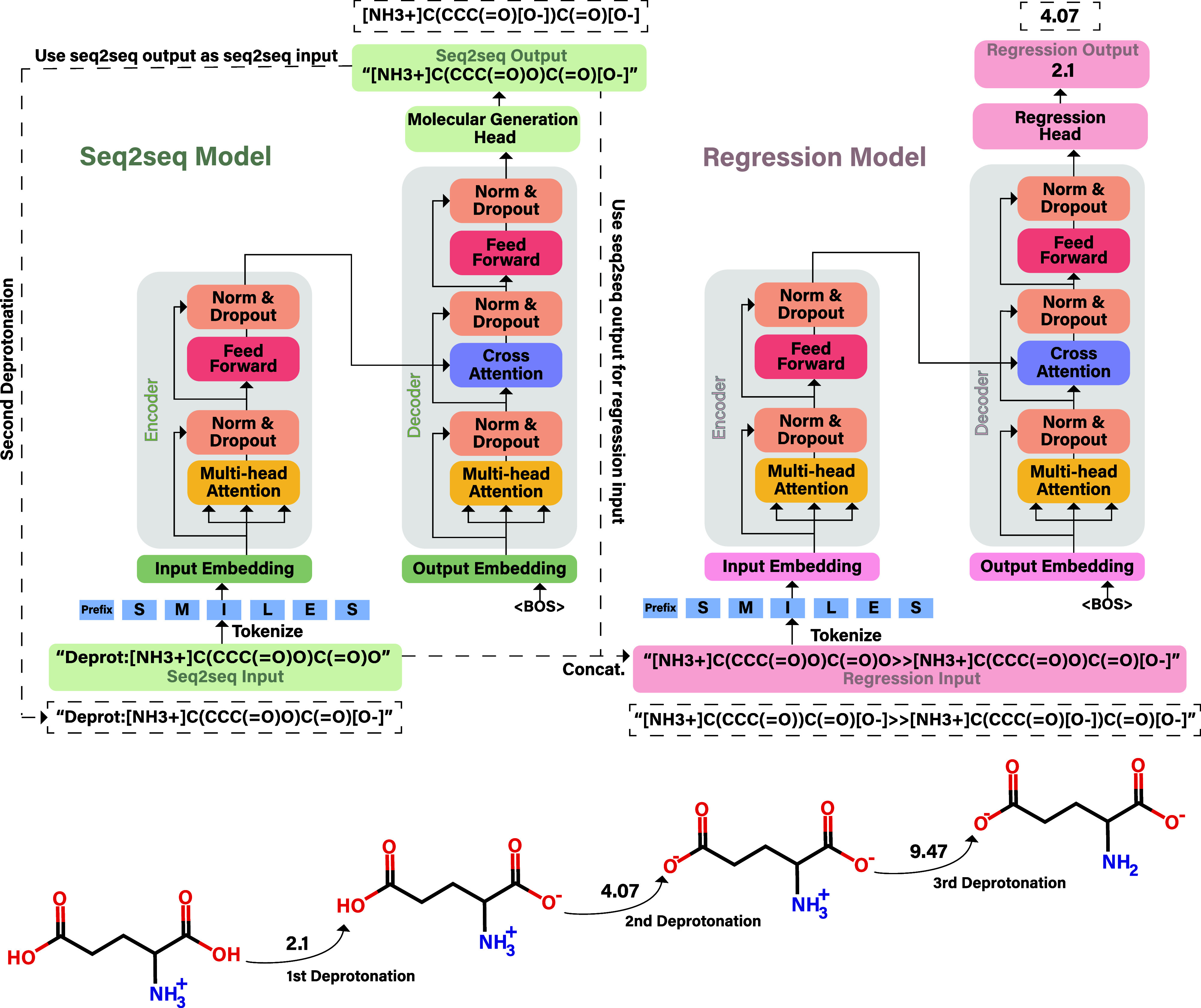
Generalization of the inputs and outputs of T5pKa. The sequence-to-sequence task highlighted in green is a unified model, where the deprotonation and protonation of a molecule are predicted with the use of either the “Prot”: or “Deprot”: prefixes along with a SMILES string representation of a molecule. There is an option to combine the inputs and outputs from the sequence-to-sequence model with “≫” and use this as an input for the microscopic p*K*_a_ model (seen in pink). We add a regression head to the end of the model to get the microscopic p*K*_a_ predictions. Consistent with experimental titration behavior, the sequence-to-sequence model is capable of using its own predictions as inputs to predict the subsequent ionization state for a given polyprotic molecule. Then, we can concatenate the sequence-to-sequence model input with the sequence-to-sequence model output for the regression model, and we can receive the predicted p*K*_a_. The dotted boxes illustrate the next predicted ionization step for this molecule.

## Method and Materials

### T5pKa Model

T5pKa employs a T5 model, which shares key elements with the vanilla transformer architecture, including an encoder-decoder in which input sequences undergo tokenization that separates the sequence into smaller pieces called “tokens.” These tokens are embedded and processed by the encoder using self-attention and feed-forward layers with layer normalization. Subsequently, the decoder uses autoregressive attention to produce the probability of each outcome. The distinction between the conventional transformer architecture and T5 resides in both the inclusion of relative position embeddings and the placing of the layer normalization outside of the residual path in T5. Additionally, T5 has a task-specific (text) prefix that is prepended to the input to help the model distinguish between different text-to-text tasks.^31,35^ Uniquely within the original T5Chem model, it incorporates an additional regression and classification head that offers the flexibility to undertake a regression or a classification task. As done in the original paper, the regression task implements a soft-label strategy for training labels normalized by min–max scaling. The regression head is a single linear layer with an output dimension of two, and it represents weights for the minimum and maximum values. Training uses the Kullback–Leibler divergence loss to minimize the difference between the predicted and target label distributions. For the sequence-to-sequence tasks, the traditional T5ForConditional output layer from HuggingFace^37^ transformers is called, which is referred to as the molecular generation head in the paper.^36^

In this work, we trained a sequence-to-sequence (seq2seq) or text-to-text task model that predicts the ionized form of a molecule in canonical Simplified Molecular Input Line Entry System (SMILES)^16^ format using RDKit’s^38^ package (version 2022.03.2). Furthermore, we use T5Chem’s architecture to develop regression models capable of predicting microscopic p*K*_a_ based on previously determined microstates. Our approach utilizes character-level tokenization, where each character within the SMILES string represents a token assigned a specific value. As a result, stereochemical information is implicitly encoded through the SMILES syntax generated by RDKit. In particular, symbols “@”, “\”, and “/” represent chiral centers and cis/trans configurations, respectively. Notably, the prefix remains uncharacterized, and a single value is assigned per prefix. The differentiation of prefixes and the flexibility of adding special tokens within T5Chem facilitates the selection of an appropriate output layer and task distinction. In T5pKa, we introduce p*K*_a_ task-specific prefixes tailored to our models. The prefixes “Prot” and “Deprot” are reserved for the sequence-to-sequence model to distinguish between a protonation or deprotonation task during multitasking training (Figure 1). During the microscopic p*K*_a_ regression task, the prefix “Pairs” is automatically prepended to all inputs, as it is a single-task model. Lastly, we added a task named “micropka”, which uses the regression head of the T5Chem model and works just as the regression task seen in the original paper. For more in-depth implementation and model details, we refer the reader to the original T5Chem paper^36^ and to the Supporting Information to reduce redundancy.

### Data Set Preparation

Recent discussion by Zheng et al.^11^ on the lack of consistency in terminology used in p*K*_a_ data as well as the need for a large experimental data set containing aqueous p*K*_a_ values inspired the creation of the pKaCHU (p*K*_a_ data that are Combined, Honed, and Updated) data set. We followed their recommendations to provide a clear representation of ionization for the molecules, especially for zwitterion and polyprotic molecules. Additionally, p*K*_a_ experiments can be performed in various solvents, but this change in conditions can alter the measured p*K*_a_ of any molecule.^13^ Consequently, we systematically compiled aqueous p*K*_a_ data from multiple published sources to construct the pKaCHU data set.

Our data set includes data from the following papers: Machine Learning Meets p*K*_a_,^22^ Comparison of the accuracy of experimental and predicted p*K*_a_ values of basic and acidic compounds (Settimo et al.),^39^ D2A-p*K*_a_ ^29^ and the data sets described by Johnston et al.^40^ The external test sets^40^ comprise data from AstraZeneca (AZ), Vertex, Manchester, Morgen-Thaler, and the Comparison of Nine programs for p*K*_a_ prediction.^41^ Together these external test sets allocated 694 new p*K*_a_ values into our data set before the data cleaning. Settimo et al.'s paper allowed us to add on 125 more molecules, while D2A-p*K*_a_ contributed 2875 p*K*_a_ values in aqueous solution extracted from the iBond database.^42^ Other models, like the holistic p*K*_a_ prediction model (HM),^43^ have trained and analyzed on the values from the iBond database and performed well. In addition, we added 40 p*K*_a_ values from amino acids (Figure 2). Lastly, Reaxys[Fn fn1] ^,^ ^44^ and IUPAC^45^ data served as a reference benchmark for cross-validation. Specifically, IUPAC molecules were matched against entries in the other data sets, and only overlapping molecules were retained. Altogether, this integration yielded 9748 p*K*_a_ values prior to data cleaning.

**Figure 2:**
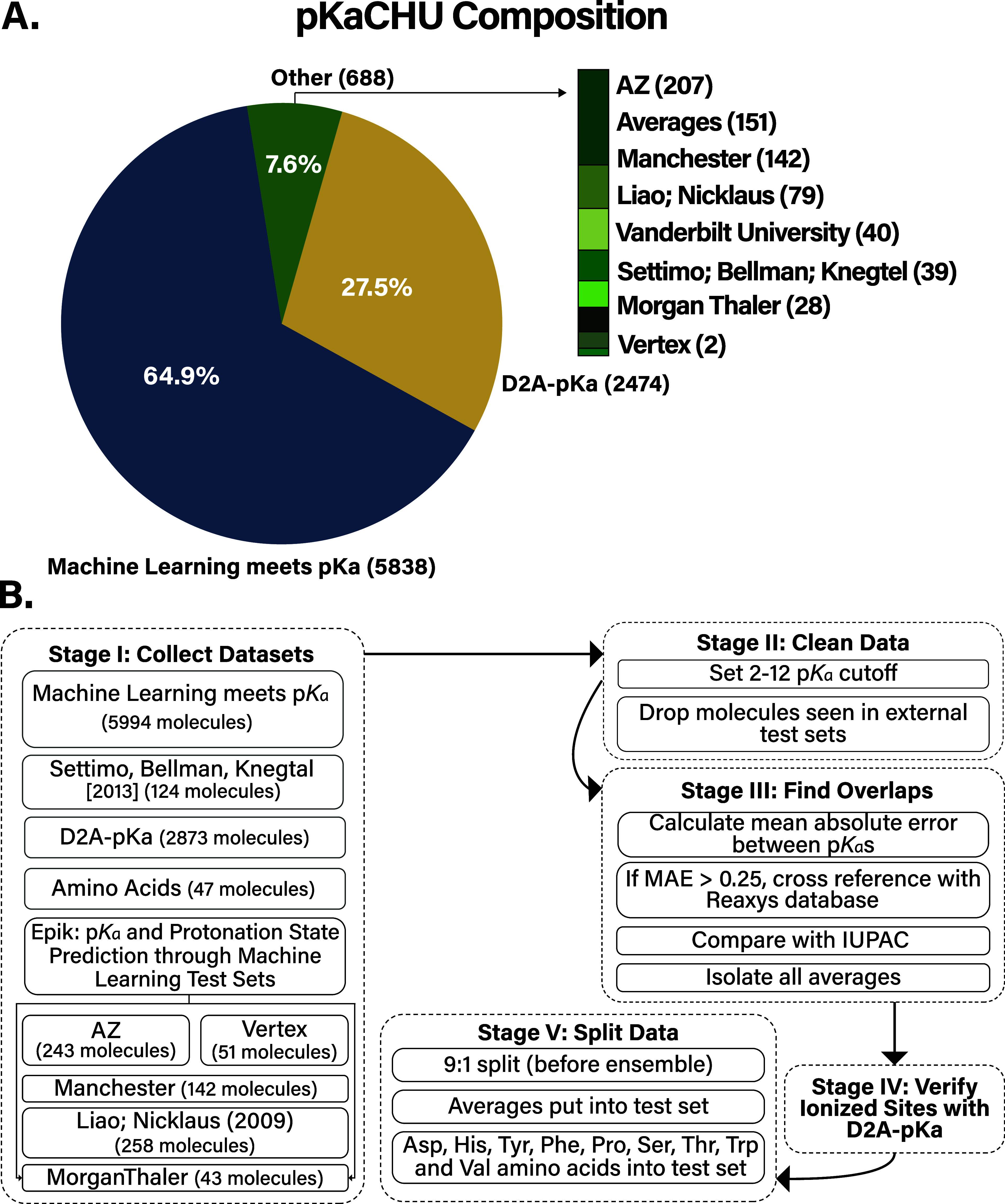
Composition and curation workflow for pKaCHU. (A) Number of curated microscopic p*K*_a_ entries contributed by each source after standardization and overlap resolution. (B) Curation pipeline: compilation from multiple sources; structure and charge standardization; filtering to aqueous measurements within a defined p*K*_a_ range; resolution of overlapping records (exact matches retained; near-matches averaged); assignment/validation of ionization-site annotations; and splitting into training, validation, and test sets.

During curation, we focused on resolving overlaps across data sets. Any discrepancies in overlapping entries were verified using Reaxys,^44^ which ensures that the reported p*K*_a_ values correspond to measurements in aqueous solution at 25 °C. When duplicate entries had consistent measurements (difference ≤0.5 p*K*_a_ units), we computed the average p*K*_a_, which was subsequently reserved for the test set. For overlapping molecules with larger discrepancies (difference >0.5), the most appropriate p*K*_a_ value was selected based on Reaxys^44^ confirmation. The resulting curated data set contained 9000 microscopic p*K*_a_ entries.

Finally, we applied a p*K*_a_ range cutoff of 2–12, which mirrors the natural range of biological p*K*_a_ values^46^ and the cutoff of the largest data set within pKaCHU.^22^ As pKaCHU primarily builds upon the data set of Baltruschat and Czodrowski's data (Figure 2), we extended a similar process of using ChemAxon’s Marvin p*K*_a_ calculator^47,48^ to identify ionization centers across all additional data sets except D2A-p*K*_a_, which provided its own ionization annotations. To prevent benchmark leakage, molecules originating from external test sets were subsequently removed from the training corpus to ensure consistency and nonoverlap between training and evaluation data. In addition, near-duplicate structures with external test sets were filtered out by applying a Tanimoto similarity threshold of 0.85, which further ensured that highly similar compounds were not used for training. Figure 2 summarizes this curation workflow.

To further characterize pKaCHU, we analyzed its structural diversity, ionization chemistry, and prevalence of monoprotic versus polyprotic entries. From the total of 9000 entries, pKaCHU contains 8881 unique molecular structures after RDKit canonicalization. Furthermore, a Bemis-Murcko scaffold^49^ analysis identified 2118 unique scaffolds. At the ionization-event level, nitrogen- and oxygen-centered ionization dominated the data set, together accounting for approximately 95% of the entries. Consistent with this distribution, amines, carboxylic acids, pyridine-like nitrogens, and phenols are among the most frequently occurring functional group classes. Lastly, pKaCHU is primarily composed of 8766 monoprotic entries, and 234 are polyprotic entries.

Together, these statistics indicate that pKaCHU spans diverse scaffolds and local ionization environments while remaining dominated by nitrogen- and oxygen-centered chemistry. This makes it a useful resource for training and evaluating p*K*_a_-prediction models.

### Training Details

Inspired by the approaches of Wu et al.^25^ and Mayr et al.,^24^ we adopt a two-stage training strategy for both sequence-to-sequence and regression models. In the first stage, we apply chemically informed transfer learning on a large-scale molecular data set derived from ChEMBL^50^ containing computed p*K*_a_ values. In the original T5 paper,^35^ transfer learning helped the model learn general-purpose representations that improved performance on downstream tasks. The sequence-to-sequence model is pretrained using a reaction prediction task (USPTO_500_MT).^36^ Proton dissociation can be framed as a reaction process, which makes the USPTO_500_MT reaction prediction model perfectly suited for the ionization task in our p*K*_a_ prediction setting. The regression model is pretrained using a multitarget regression (MTR) task on 123 RDKit^38^ molecular descriptors. Similar to the p*K*_a_ regression task, this involves learning structure–property relationships through regression. This elaborate pipeline significantly improves results relative to training from scratch (Tables S2 and S3). In the regression setting, pretraining consistently lowers RMSE across all external test sets, and the full pipeline yields the best overall predictive performance. For the sequence-to-sequence task, the same trend is observed: increased accuracy and a pronounced reduction in invalid predictions. Together, these results show that chemically informed pretraining contributes meaningfully to microstate and microscopic p*K*_a_ predictions.

In the second stage, the calculated p*K*_a_ data model serves as a pretrained model for the fine-tuning on the pKaCHU data set. Given the scarcity of high-quality experimental p*K*_a_ data, we found that this two-step framework allows the model to first establish a robust mapping between molecular text representations and calculated p*K*_a_ values using large unlabeled data sets, before refining its understanding on a smaller and high-fidelity experimental data set (Figure 3).

**Figure 3:**
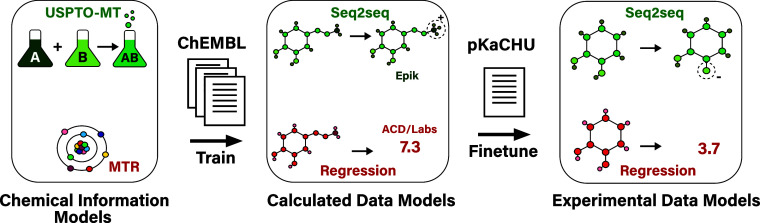
We perform a two-stage pipeline for both the sequence-to-sequence model and the regression models. First, we use chemical information models, which were described earlier as USPTO_500_MT and multitarget regression (MTR). USPTO_500_MT contains reaction prediction information for the sequence-to-sequence models, and MTR contains 123 molecular descriptor information for the regression models. We use the pretrained models for their respective tasks to train on the ChEMBL data set. Using ChEMBL and Schrodinger’s EpiK, we extract calculated p*K*_a_ information. Second, we finetune on the pKaCHU data set that contains 9000 experimental p*K*_a_ values.

In both stages, we trained an ensemble model for the regression task by using different splittings and averaging the results from each model.

Both the sequence-to-sequence and regression models contain approximately 14.7 million trainable parameters and were trained on an NVIDIA A100 GPU. Full hyperparameter, computational, and pretraining details are provided in the Supporting Information.

### Learning p*K*_a_ Prediction

Previous studies demonstrate pretraining as an effective way to improve the performance of a model.^51–55^ Here, we extracted approximately 1.8 million molecules from the ChEMBL (ver.25) database. ChEMBL is a large and open-access database containing chemical, bioactivity, and genomic data.^50^ The version of ChEMBL that we use lists physicochemical properties that were calculated by Advance Chemistry Development, Inc. (ACD/Laboratories).^56,57^ We use the p*K*_a_ values calculated using ACD/Laboratories, and we find their corresponding ionizable sites through Schrodinger’s Epik 2021.^18^ After cleaning the data, we ended with a total of approximately 1.31 million molecules to train for the calculated p*K*_a_ model. One model was trained to predict ionizable sites given by Epik, and one model was trained to predict the calculated p*K*_a_ values from ACD/Laboratories, as given by the ChEMBL database. By training on a large amount of molecules, the finetuned models can gain insight on the mapping between molecular text-based input and p*K*_a_ information before training on a more refined data set like pKaCHU. Lastly, we finetune on the newly introduced dataset, pKaCHU, that contains 9000 experimental p*K*_a_ values.

### Ensemble Model

For the microscopic p*K*_a_ regression, the reported results (Table 2) are derived from an ensemble of 10 independently trained models that shares the same architecture and hyperparameters. The ensemble was constructed to improve robustness and reduce the dependence on any single split. Specifically, five ensemble members were trained using random splits, and five were trained using scaffold-based splits. The final ensemble predictions were obtained by averaging the outputs of all of the 10 models.

**Table 1:** Top-1 Microstate Prediction Accuracy and Microscopic p*K*_a_ RMSE on the External Benchmark Datasets^a^

	data size	top-1 accuracy ↑	given inputs ↓	predicted inputs ↓
novartis	280	93.6%	0.967	0.993
literature	123	100%	0.513	0.513
SAMPL6	10	100%	0.865	0.865
SAMPL7	20	100%	0.805	0.805
SAMPL8	25	96.0%	0.842	0.815

a “Given inputs” denotes regression performance using dataset-provided microstate pairs, whereas “Predicted inputs” denotes regression performance using microstate pairs generated by the sequence-to-sequence model.

**Table 2:** External Test-Set Performance (RMSE in p*K*_a_ Units; Lower is Better) for Microscopic p*K*_a_ Prediction Across Benchmark Datasets^a^

	type	novartis ↓	literature ↓	SAMPL7 ↓	SAMPL8 ↓
Epik Classical^18^	empirical	1.16	0.92	–	–
ML meets p*K*_a_ ^22^	RF	1.51	0.79	–	–
pkasolver-epic^24^	GNN	0.93	0.82	–	–
pkasolver-light^24^	GNN	1.13	0.82	–	–
Graph-p*K*_a_ ^8^	GNN	–	–	0.93	1.23
H-SPOC^28^	XGBoost	–	–	0.71	**0.56**
Uni-p*K*_a_ ^27^	GT	0.84	–	**0.60**	0.88
MolGpKa^23^	GNN	1.18	1.04	0.98	1.54
Marvin^19^	empirical	1.21	0.87	0.94	0.69
Qup-kake^17^	GNN/QM	**0.79**	0.54	0.85	1.04
T5pKa	T5	0.97	**0.51**	0.81	0.84

a T5pKa results are compared with representative commercial tools and published machine-learning models; values for published methods are taken from corresponding original publications when not rerun here.

For the calculated p*K*_a_ data set, a 9.5:0.5 split was applied, and the larger portion was further divided into training and validation subsets through cross-validation using either random or scaffold-based splitting. For the experimental pKaCHU data set, a 9:1 split was used, with the larger portion partitioned similarly to the calculated data set. In all cases, the test sets from both the calculated data set and pKaCHU were excluded from model training and were not seen by any of the ensemble members.

To assess variability across ensemble members, we evaluated each individual model on the internal test set, as shown in Table S4. The models achieved MAE values ranging from 0.491 to 0.567 and RMSE values from 0.788 to 0.959, and the ensemble obtained an MAE of 0.438 and an RMSE of 0.757. On the external benchmarks, random-split and scaffold-based split models demonstrated similar performance, as shown in Table S5. For more information on the model and its training details, please refer to the Supporting Information.

## Results and Discussion

### Evaluation

To evaluate the model performance, we tested T5pKa on five external test sets. The test sets referred to are Novartis, a literature compilation (called Literature here) created by Baltruschat and Czodrowski,^22^ SAMPL6,^6^ SAMPL7,^58^ and SAMPL8 ^59^ data sets.

### Unifying Predicted Ionizable Sites with a Microscopic p*K*_a_ Prediction Model

A key feature of T5pKa is that ionization site identification is formulated as a sequence-to-sequence task, while microscopic p*K*_a_ prediction is performed with a regression model operating on microstate pairs. Because both components use a shared text-based molecular representation, predicted microstates from the sequence-to-sequence model can be passed directly into the regression model. This design explicitly couples microstate generation to microscopic p*K*_a_ estimation, enabling an integrated end-to-end workflow.

Table 1 reports the top-1 accuracy of the ionization-site prediction model and the root-mean-square error (RMSE) of the p*K*_a_ prediction model on the external test sets. Here, Top-1 accuracy refers to the fraction of molecules for which the first ionized SMILES prediction generated by the sequence-to-sequence model exactly matches the reference target. For the regression model, “Given inputs” reports performance using the data set with provided microstate pairs, whereas “Predicted inputs” reports performance when the regression model uses microstate pairs generated by the sequence-to-sequence model.

From Table 1, T5pKa excels in predicting the ionization sites of a given molecule. Across all external test sets, the model achieves greater than 93% top-1 accuracy for predicting the correct protonated/deprotonated atom, including perfect ionization prediction from the literature, SAMPL6, and SAMPL7. In particular, the SAMPL8 data set (96% top-1 accuracy) contains only a single mismatched prediction.

On the Novartis data set, T5pKa achieved a Top-1 accuracy of 93.6%. The analysis of the mismatched cases indicates that most errors involve nitrogen ionization sites. These incorrect assignments predominantly occur in heterocyclic systems, where the model occasionally predicts protonation at a neighboring nitrogen atom within the same or an adjacent ring. The predicted sites remain chemically plausible, suggesting that these discrepancies may arise from competing basic centers with similar proton affinities. In some cases, this behavior is associated with the presence of tautomeric effects. A representative example is observed in histidine, as the model assigns protonation to the alternative nitrogen in the imidazole ring during the second ionization step (see the Supporting Information). Many of these mismatches were recovered in the Top-2 accuracy predictions, increasing the accuracy to 97.1% and indicating that the correct ionization site is typically ranked among the top two predictions.

The SAMPL6 data set contains ten molecules, two of which were experimentally analyzed with NMR spectroscopy. The remaining eight molecules are derivatives of those two parent molecules, so their ionization sites are inferred from the experimentally characterized compounds.^6^ As shown in Figure S11, these ten molecules exhibit two possible tautomeric forms. We evaluated these tautomers using sPhysNet-Taut^60^ to determine their relative stability, and the more stable tautomers were subsequently used as model inputs (Table S8). With this protocol, T5pKa was able to predict the most stable ionized form without explicitly training for tautomer handling. Additionally, this external data set contains one example of a sequential microscopic p*K*_a_ (SM14), which T5pKa was also able to predict correctly.

Consequently, the ionization states predicted by the sequence-to-sequence model can therefore be used as inputs to the regression model for p*K*_a_. For the Novartis data set with less than 100% top-1 accuracy, the RMSE increases slightly when predicted rather than when reference microstates are used. In contrast, for SAMPL8, the use of predicted inputs slightly improves performance. Overall, this result supports the utility of the unified framework for molecules whose ionizable sites are not known a priori and enables fully automated microscopic p*K*_a_ prediction directly from the molecular structure alone.

The T5pKa regression model is competitive with the existing p*K*_a_ prediction methods. From Figure 4, T5pKa demonstrates strong predictive accuracy with all predicted p*K*_a_ values clustering closely to the line of best fit, which indicates good agreement between actual and predicted values. This is further supported by *R* ^2^ values ranging from 0.82 to 0.95, indicating strong linear correlation across all five data sets. T5pKa excels on the literature data set, likely due to this data set containing a clear distribution of ionized atom types across a broad range of p*K*_a_ values. Regardless, T5pKa maintains strong performance on data sets with more of a mixed relationship between ionized atom type and p*K*_a_. Additionally, we performed bootstrap resampling on each external test set and recalculated the RMSE over the resampled data sets to assess the stability of these results. T5pKa has stable performance on the larger Novartis and Literature data sets, with a 95% bootstrap confidence interval (CI) of 0.88–1.06 on RMSE of 0.967 and a 95% bootstrap CI of 0.43–0.59 on RMSE of 0.513, respectively. For the smaller SAMPL benchmarks, the RMSE values were 0.865 (95% bootstrap CI: 0.43–1.30) for SAMPL6, 0.805 (95% bootstrap CI: 0.60–0.99) for SAMPL7, and 0.842 (95% bootstrap CI: 0.61–1.05) for SAMPL8. The wider intervals observed for the SAMPL data sets are consistent with the increased variability expected from the small sample size of the data sets. Overall, these results place T5pKa within the range of current state-of-the-art microscopic p*K*_a_ models (Table 2).

**Figure 4:**
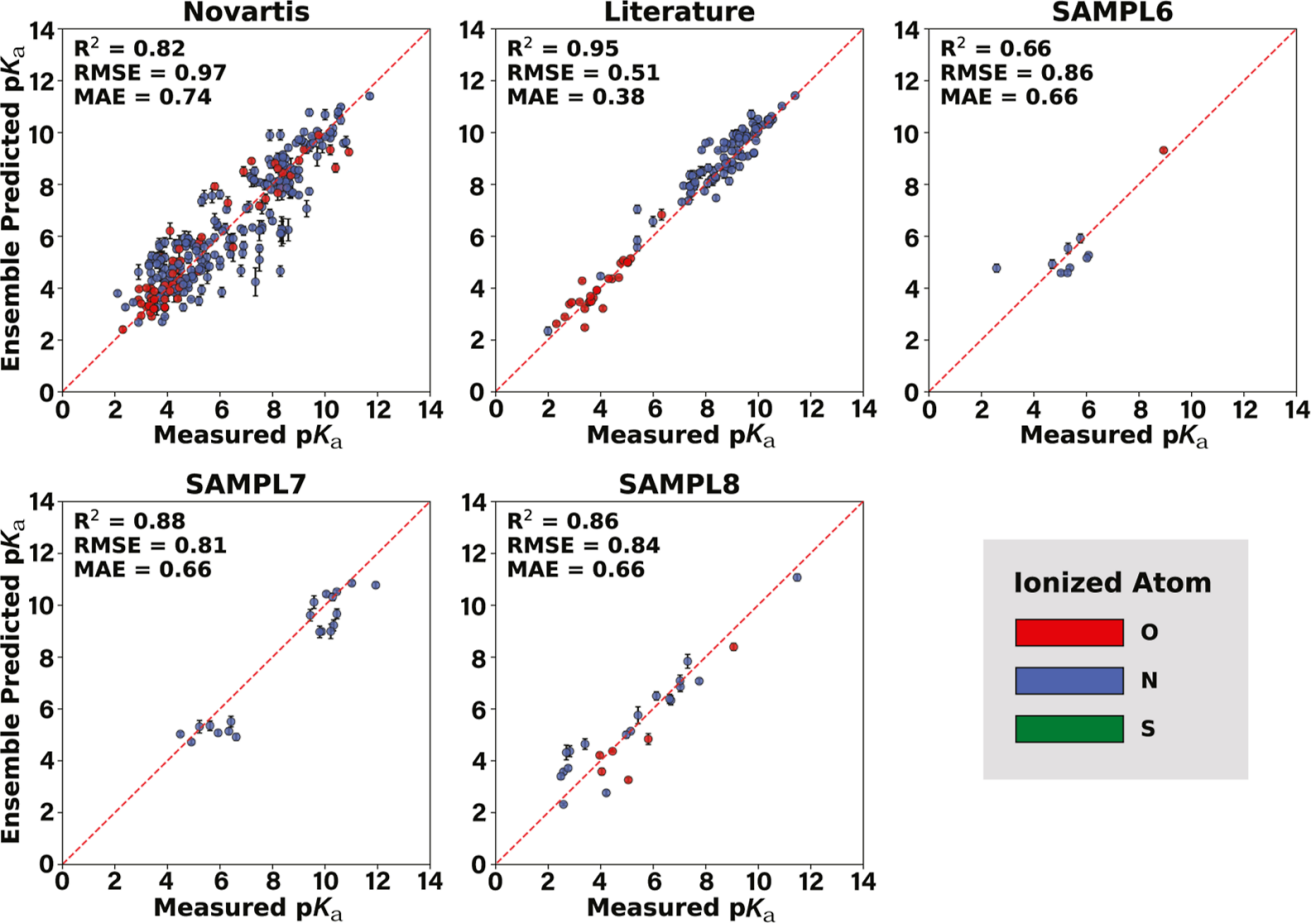
Scatter plots with the performance of T5pKa for each external test set: Novartis, Literature, SAMPL6, SAMPL7, and SAMPL8. We test the microp*K*_a_ model, and we observe good performance among all five plots. Error bars indicate the standard deviation across ensemble predictions and points colored by ionized atom type.

In Table 2, T5pKa consistently ranks among the top four models in performance. Notably, it achieves the best reported performance on the literature data set, with an RMSE of 0.51 for the microscopic p*K*_a_ task. On the Novartis, SAMPL7, and SAMPL8, T5pKa’s performance is comparable to the other models. The SAMPL6 data set is not included in this comparison because our evaluation uses two molecules from NMR experiments and their derivatives, which have not been tested by other programs, so it prevents a fair benchmark comparison.

To further assess the performance on molecules with multiple ionizable sites, we evaluated the models on the polyprotic molecules in the internal test set. The model achieves an RMSE of 1.04 with an MAE of 0.56 on this subset, indicating reasonable generalization to multistep ionization behavior.

Although accuracy decreases for higher-order ionization steps, the overall ionization sequence remains well-preserved, with a top-1 sequence-to-sequence accuracy of 92%. These results support the applicability of the framework to polyprotic systems while also indicating that larger experimental data sets containing higher-order ionization events would likely further improve performance.

### SHAP Explanation

We applied SHAP (SHapley Additive exPlanations)^61^ to phenol, 2,4-Dinitrophenol, and a series of *N*-methylpiperidinium derivatives to examine the chemical knowledge accumulated by our model. SHAP operates on a game-theoretic approach to reduce the esoteric nature of a model’s prediction. It assigns positive or negative contributions to individual input features relative to a base value, defined as which is the average model prediction based on the training set. In this case, the input features are the characters in the textual input. By clustering atoms into functional groups with the EFG package and summing each group’s contribution, we gained insight into how different moieties influence p*K*_a_.

For phenol and 2,4-dinitrophenol, the T5pKa ensemble predicted p*K*_a_ estimates in close agreement with experimental values: 10 (9.98 experimentally^62^) for the phenol molecule and 3.8 (4 experimentally) for the 2,4-dinitrophenol molecule. Figure 5A depicts a positive contribution from the benzene ring and a small net negative contribution on the ionized hydroxyl group, leading to a p*K*_a_ of 10. In contrast, the nitro substituents in 2,4-dinitrophenol contribute negatively toward the base value of 6.48. This is consistent with nitro groups lowering the basicity of a molecule,^63^ so the two nitro groups added to the original phenol molecule will lower the p*K*_a_ value of the molecule. We further examined the effect of alkyl chain length on the predicted p*K*_a_ for a series of *N*-methylpiperidinium derivatives. Experimentally, longer alkyl chains are associated with higher p*K*_a_ values, and this trend is simultaneously reflected in SHAP analysis (Figure 5B). The carboxylic acid group showed less negative contributions with increasing chain length, the charged nitrogen remained consistently negative, and the methyl group contains a slightly positive contribution throughout but remains mainly unchanged across the series. The charged nitrogen fragment remains consistently negative, but its contribution is partially offset by the positive piperidine-ring carbons. This signifies that the increase in predicted p*K*_a_ across the series is primarily associated with the increasing positive alkyl-chain contribution. Similar substituent-dependent effects were observed for pyrrolizidine derivatives, where replacing hydroxyl or fluorinated substituents with a methyl shifted predictions toward higher p*K*_a_ values^64^ (Figure S10). Together, these results indicate how influential added functional groups can be on the overall p*K*_a_, as well as the ability of our model to gain chemical intuition.

**Figure 5:**
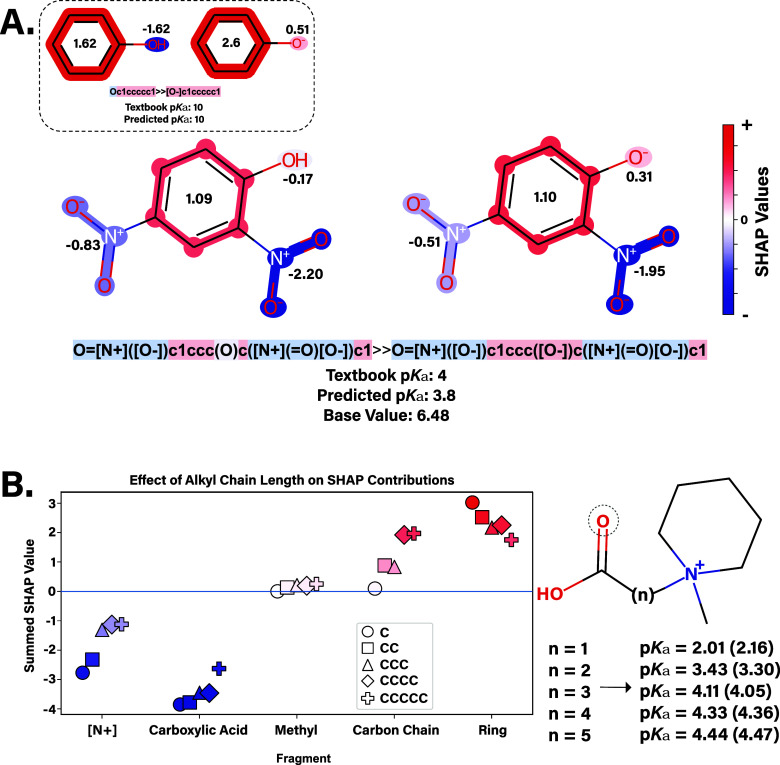
(A) SHAP analysis on phenol and 2,4-dinitrophenol. The more red the functional group, then the greater the contribution to the overarching prediction (base value). The reverse is true for the blue-toned functional groups. In this example, we can see once the nitro groups were added to the molecule, then there was a decrease in the p*K*_a_ prediction, helping it get to the correct prediction. (B) Effect of alkyl chain length on predicted p*K*_a_ values using SHAP contributions as explanation. A series of *N*-methylpiperidinium derivatives is evaluated with increasing chain length (*n* = 1–5). The molecule’s experimental p*K*_a_ increases with n, and the predicted values (in parentheses) are in agreement. The plot shows the sum of SHAP values based on functional fragments: a charged nitrogen atom, a carboxylic acid, a methyl group, an alkyl chain, and a ring system. Blue indicates a negative SHAP contribution, while red indicates a positive SHAP contribution.

## Conclusion

In this work, we introduced T5pKa, a sequence-based framework that unifies microstate enumeration and microscopic p*K*_a_ prediction within a shared text-based modeling pipeline. T5pKa offers several practical advantages: it predicts ionization microstates and microscopic p*K*_a_ values within a common representation, unifies protonation and deprotonation prediction into one model, reduces reliance on manually specified ionization rules, and benefits from transfer learning on large-scale chemical language model tasks. We further introduced the pKaCHU data set, a curated microscopic p*K*_a_ data set designed to provide clearer ionization annotations and more consistent experimental conditions for model development. Together, these contributions enable competitive performance for both ionization-site prediction and microscopic p*K*_a_ regression across external benchmark data sets. The added interpretability analyses further suggest that chemically meaningful features can be learned from one-dimensional molecular representations. Although T5pKa performs well on monoprotic systems and shows reasonable generalization to polyprotic molecules, higher-order ionization steps remain more challenging. Future work will therefore focus on expanding the coverage of experimental polyprotic data and incorporating a more explicit treatment of tautomerization effects to further extend the applicability of the framework.

## Supplementary Material



## Data Availability

Data and Software Availability: the data sets and source codes used in this work are readily available for use. The experimental p*K*_a_ data can be found in https://zenodo.org/records/20089807. Instructions for creating the calculated p*K*_a_ data can be found in https://github.com/charlotteinfante/t5pKa-data because generation of Epik-based labels requires a Schrödinger license. The source code for model training and model evaluation configuration files are available at https://github.com/charlotteinfante/t5pKa. The trained checkpoints are available at https://huggingface.co/charlotteinfante/t5pka_checkpoint/tree/main.

## Footnotes

a Copyright © 2022 Elsevier Limited except certain content provided by third parties. Reaxys is a trademark of Elsevier Limited.

## Notes

Software and implementation details, molecular preprocessing workflow, pKaCHU data set characterization, training protocols, transfer-learning strategy, additional benchmarking and ensemble analyses, additional SHAP interpretation, and SAMPL6 tautomer analysis (PDF)
