# Supplementary material for: Unifying pK a and Protonation Prediction with Sequence-Based Deep Learning
Source: J Chem Inf Model. 2026 May 29;66(12):6972–82. doi: 10.1021/acs.jcim.6c00556 (PMC13292215; doi:10.1021/acs.jcim.6c00556)
Supplement: Supplementary file 1 [file ci6c00556_si_001.pdf]

Supporting Information: Unifying pK_a and Protonation Prediction with Sequence-based Deep Learning

Charlotte Infante,[†] Jieyu Lu,[†] Xiaolin Pan,[†] Song Xia,[†] and Yingkai Zhang[*,†,‡,¶]

†*Department of Chemistry, New York University, New York, NY 10003, United States*

‡*Simons Center for Computational Physical Chemistry at New York University, New York, NY 10003, United States*

¶*NYU-ECNU Center for Computational Chemistry at NYU Shanghai, Shanghai 200062, China*

E-mail: yingkai.zhang@nyu.edu

Contents

Implementation **S3**

Dataset Details **S3**

Training Details **S6**

Pretrained Models for Transfer Learning **S11**

Model Performance and Ensemble Analysis **S13**

SAMPL6 NMR molecules **S21**

Implementation

Echoing the implementation from the original T5Chem model, we use Python (version 3.9.13), and we process all molecular data using RDKit's package (version 2022.03.2). The same versions from Hugging Face transformers (version 4.10.2) and PyTorch (version 1.7.1) were used as in the original paper.

Dataset Details

To further characterize the dataset, we analyzed (i) the number of unique molecular structures, (ii) the count difference between monoprotic and polyprotic molecules, (iii) the distribution of ionized atoms in the training set, and (iv) the chemical diversity based on the functional groups and molecular scaffolds.

The pKaCHU dataset contains 9,000 total entries, and we found 8,881 correspond to unique molecular structures. These unique molecular structures were identified using RDKit's canonicalization of SMILES strings. Among the entries, the dataset is predominately composed of (8,766) monoprotic molecules, and 234 entries had polyprotic qualities. Within these polyprotic molecules, only a few contained three pK_a values. To ensure that we had some of these triprotic acids, we manually chose four amino acids (arginine, cysteine, glutamic acid, and lysine) to add into training. After the data processing, arginine was not present, and cysteine was reduced to two pK_a entries, but glutamic acid and lysine remained intact.

Additionally, we evaluated the diversity of the dataset by analyzing the different elements involved in the acid dissociation reaction, like the ionizable atom and the functional groups involved. Figure S1 references the different counts of atoms involved in the reaction seen in the training data. The dataset heavily favors nitrogen-based and oxygen-based ionization, while carbon and sulfur atom (de)protonations are minimal. To further understand these ionizable atoms, we looked into the chemical environment of the full dataset. Table S1 lists all

the functional groups involved in the ionization reaction. Similarly to the ionized atom count, the functional group with the highest count is the amine group. Secondly, the carboxylic acid group, which involves oxygen deprotonation, is the second most seen functional group. The following two most seen moieties were pyridine rings and phenol groups, which both contain nitrogen and oxygen atoms, respectively.

Lastly, we explored the diversity of the core structure of the molecules by running a scaffold analysis. The scaffold diversity resulted in 2,118 unique scaffolds. Given the distribution of the pK_a values as seen in Figure S2 and the diversity of the dataset, we can conclude that this dataset provides meaningful chemical diversity and coverage for pK_a prediction.

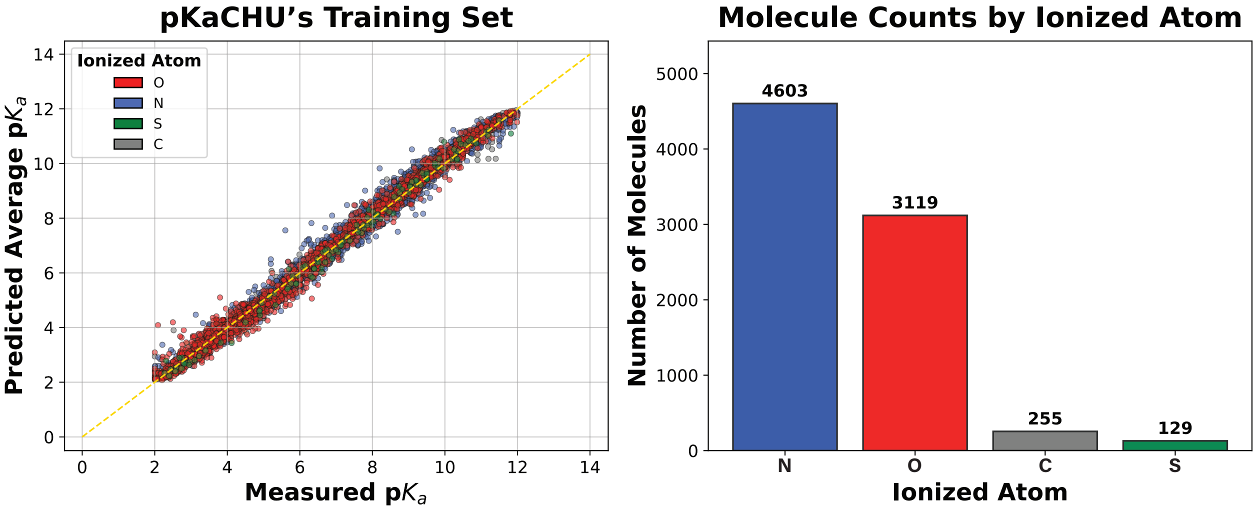

Figure S1: A scatterplot depicting the prediction of the training set that was done during the 9:1 splitting can be seen to the left. The ionizable atom for each molecule is also pointed out on the graph to see which ionizable atom is predicted better. As seen in the bar graph to the right, the data contains ionized nitrogens the most, followed by oxygens, carbons, and sulfur atoms. From this, it correlates with the better predictions of molecules containing either nitrogen or oxygen ionization.

Table S1: Distribution of ionizable functional groups in the pKaCHU dataset. Functional groups are assigned based on the atom undergoing ionization and its local chemical environment.

**Functional Group**	**Full Count**	**Ionized Atom**
Amine	3349	N
Carboxylic acid	1842	O
Pyridine-like N	1400	N
Phenol	962	O
Iminium	345	N
Alcohol	318	O
Carbon Ionization	285	C
N-oxide / N–O system	223	O
Thiol	145	S
Phosphate / phosphonate	46	O
Peroxide / O–O system	30	O
Arsonic acid	24	O
Sulfur oxyacid	11	O
Peroxy acid	9	O
Silicic acid	4	O
Cl-containing oxyacid	2	O
Selenic / selenium oxyacid	2	O
Boronic acid	1	O
Germanic acid	1	O
Hypobromous acid	1	O

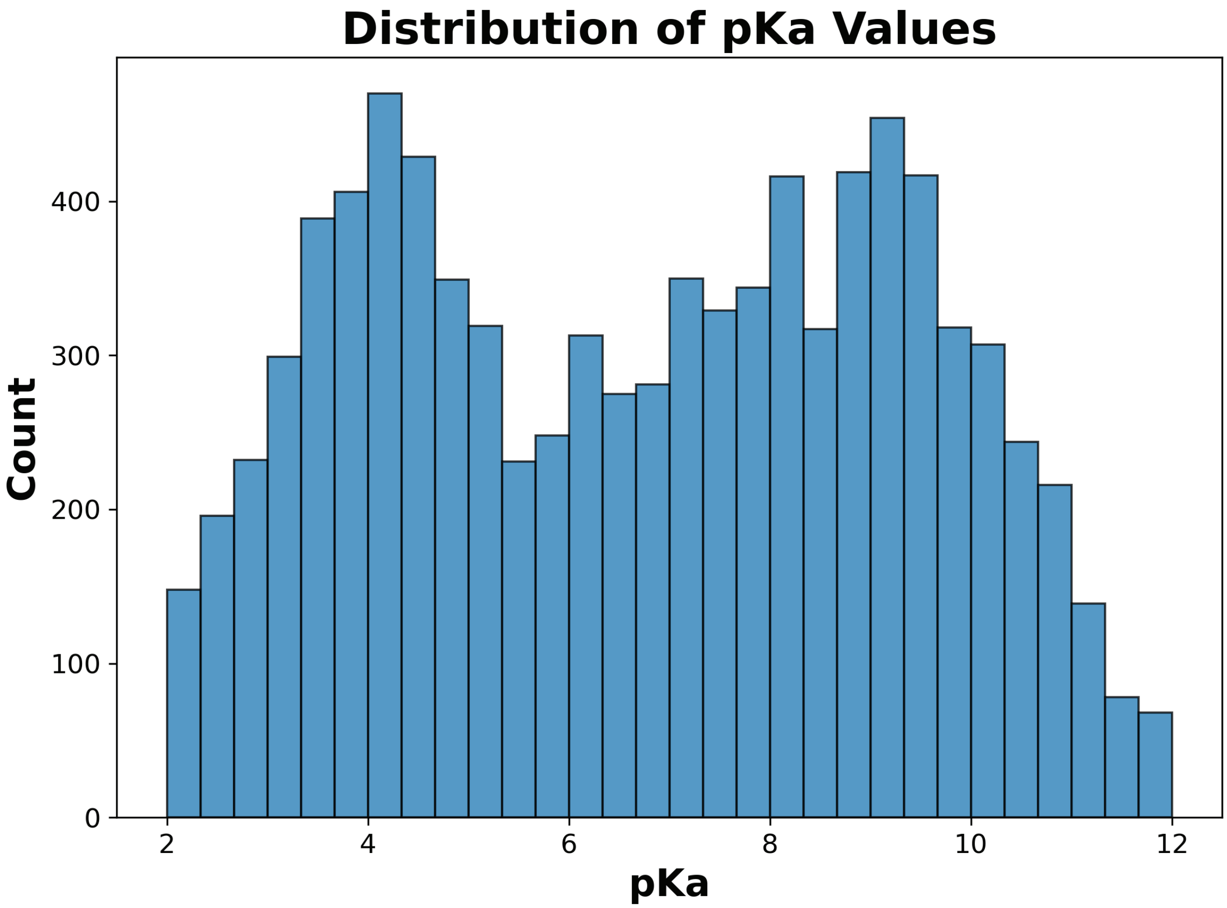

Figure S2: We highlight the distribution of pK_a values in the pKaCHU dataset. There are higher counts of pK_a values between 3-5 and 7-10.

Training Details

Hyperparameters

For the models trained on the calculated pK_a dataset, we do a 9.0:0.5:0.5 split for the sequence-to-sequence models. We use a learning rate of 6×10^{-4} with a batch size of 128 and trained for 100 epochs.

For the regression models trained on the calculated pK_a dataset, we first split 9.5:0.5, and the 95% goes into the ensemble model procedure, so there is a cross validation training taking place. We use a learning rate of 1×10^{-3} with a batch size of 128 and 100 epochs.

When finetuning on the pKaCHU dataset, the sequence-to-sequence model used a learn-

ing rate of 6×10^{-4} with a batch size of 128 and trained for 150 epochs. We performed a 8:1:1 split.

For the regression models trained on the pKaCHU dataset, we performed a 9:1 splitting, but 90% of the data would go through the ensemble model procedure where cross validation training was performed. We used a learning rate of 5×10^{-4} with a batch size of 128, and trained for 150 epochs.

For all models, training was run for a fixed number of epochs, so no early stopping was used.

Model Size and Computational Efficiency

Both the sequence-to-sequence and regression models contain approximately 14.7 million trainable parameters. All models were trained using NVIDIA A100 GPUs. Inference is computationally efficient, and it can be performed on both GPU and CPU hardware. When evaluating the inference time for prediction on GPU hardware, the regression model requires 0.009 seconds per molecule, and the sequence-to-sequence model requires 0.32 seconds per molecule.

Splitting

Ionized Splitting

We introduced another condition termed “ionized splitting”, in which molecules could be partitioned either randomly or according to their scaffolds, provided that any polyprotic molecule’s ionized forms and their corresponding pK_a values were assigned entirely to training, validation, or test set (Figure S3).

Amino Acid Splitting

During the ensemble model construction, we employed a 9:1 data split. In this configuration, we ensured that 11 amino acids were included in the training set and 9 amino acids in the test set. Specifically, the training portion comprised four amino acids with three pK_a values and seven amino acids with two pK_a values, while the remaining three amino acids with three pK_a values and six amino acids with two pK_a values were allocated to the test set.

For the sequence-to-sequence, we also ensured that the same amino acids were in the training and test set, respectively.

Ensemble Splitting

We trained five T5 models using random splits, and we trained another five T5 models using scaffold-based splits (Figure S4). Table S5 shows the results of using the ensemble average of random splitting versus the ensemble average of scaffold splitting.

Textual Representation

For the sequence-to-sequence task, the input molecule was represented using its most neutral form, and the model was trained to predict either the protonated or deprotonated microstate depending on the task prefix. This formulation was independent of the regression input pair and was intentionally designed to be more challenging, as the model had to infer the relevant ionization site and the appropriate protonation-state transformation from the neutral molecular representation.

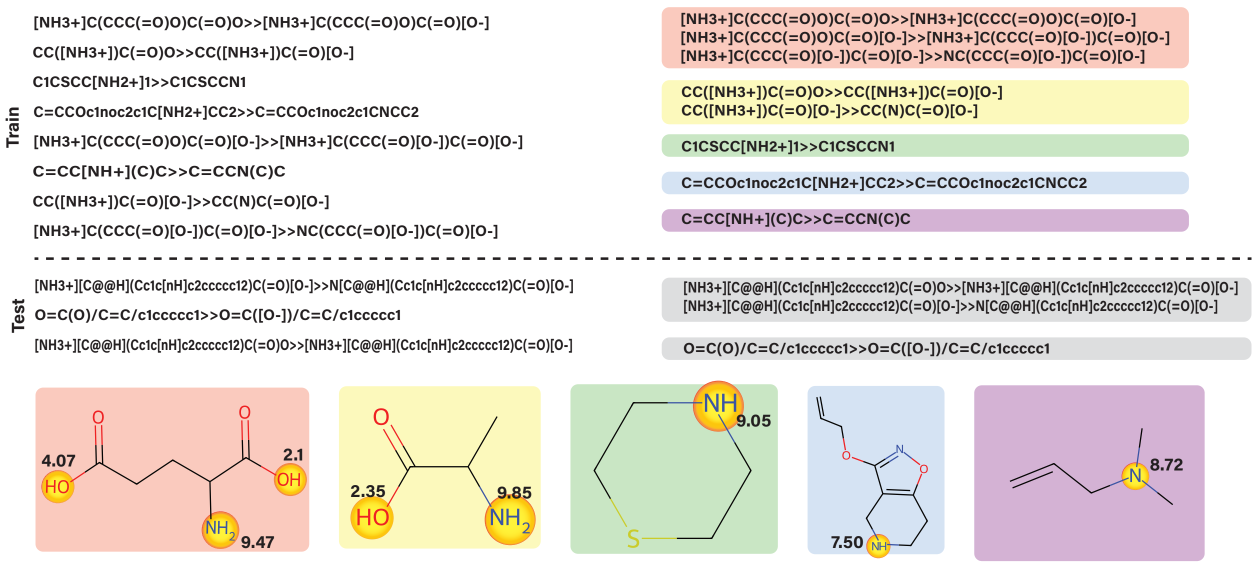

Figure S3: Ionized splitting can be described by grouping together all ionized forms of the same molecule into either training, validation, or test set. In this way, the data can still be split randomly or by scaffold, but it still can be trained on a molecule's full ionization scheme. For example, the training set has 8 molecules, but two of those molecules are polyprotic, and will be lumped together during the splitting. We can see this with the molecules in red and yellow.

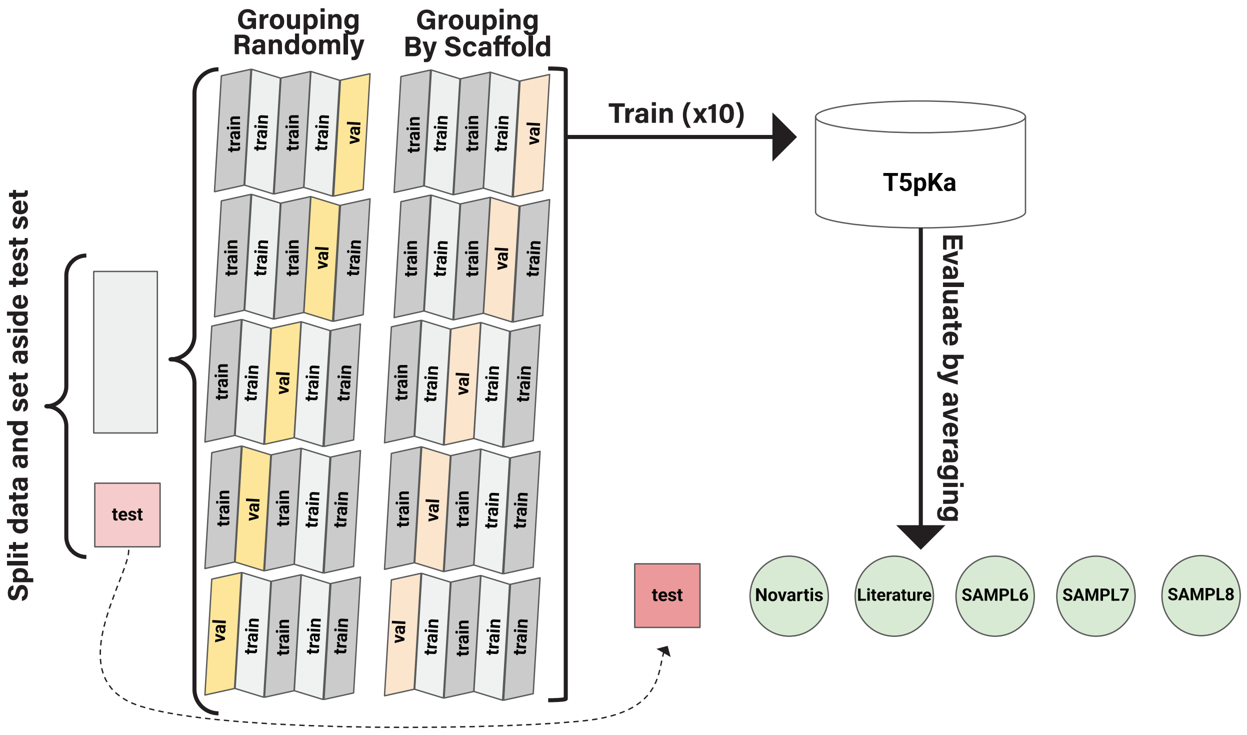

Figure S4: We show the workflow of data splitting for ensemble model. First, we save 10 percent of the data to be used as a test set. The remaining data is used in k-fold cross validation. We do this 10 times for 5 models split randomly and 5 models split based on scaffold similarity. Then, we evaluate the performance of the models by averaging the predictions, and test on 5 external test sets.

Pretrained Models for Transfer Learning

In this study, we use two models trained on related tasks to pK_a prediction. The first pretrained model is a previously trained multitasking transformer model for chemical reaction prediction (USPTO_500_MT).[1] Additionally, we introduce a second pretrained model that employs a multi-target regression objective. The transfer learning process takes place during the training of the models using a dataset containing molecules from ChEMBL[2] dataset with generated calculated pK_a values and protonation state determination from Schrödinger's Epik.

The use of USPTO_500_MT for sequence transformation tasks is motivated by framing pK_a prediction as a reaction-like process, in which ionization can be viewed as a transformation between molecular microstates. This enables effective transfer learning from diverse chemical reactions to protonation and deprotonation events. In contrast, the MTR pretrained model is well suited for regression tasks, as it introduces the model to a broad set of numerically defined molecular characteristics, facilitating efficient learning of continuous pK_a values (Figure S5).

For MTR pretraining, molecules were sourced from the PubChem database.[3] Molecular features were extracted using the RDKit[4] package, which provides 123 molecular descriptors per molecule. Afterwards, any molecules containing undefined (NaN or infinite) descriptor values were excluded. Long-tail distributions can also be seen due to a small subset of molecules that exhibit extremely large values in one or more of the 123 molecular descriptors, and these molecules are not representative of the drug-like chemical space we aim to model, so they were also removed. In the end, we concluded with a total of 94.4 million molecules. In this case, the MTR pretrained model was used for regression-based tasks, which is reflective of their respective inductive biases. Lastly, we include the impact of using pretrained models in Tables S2 and S3.

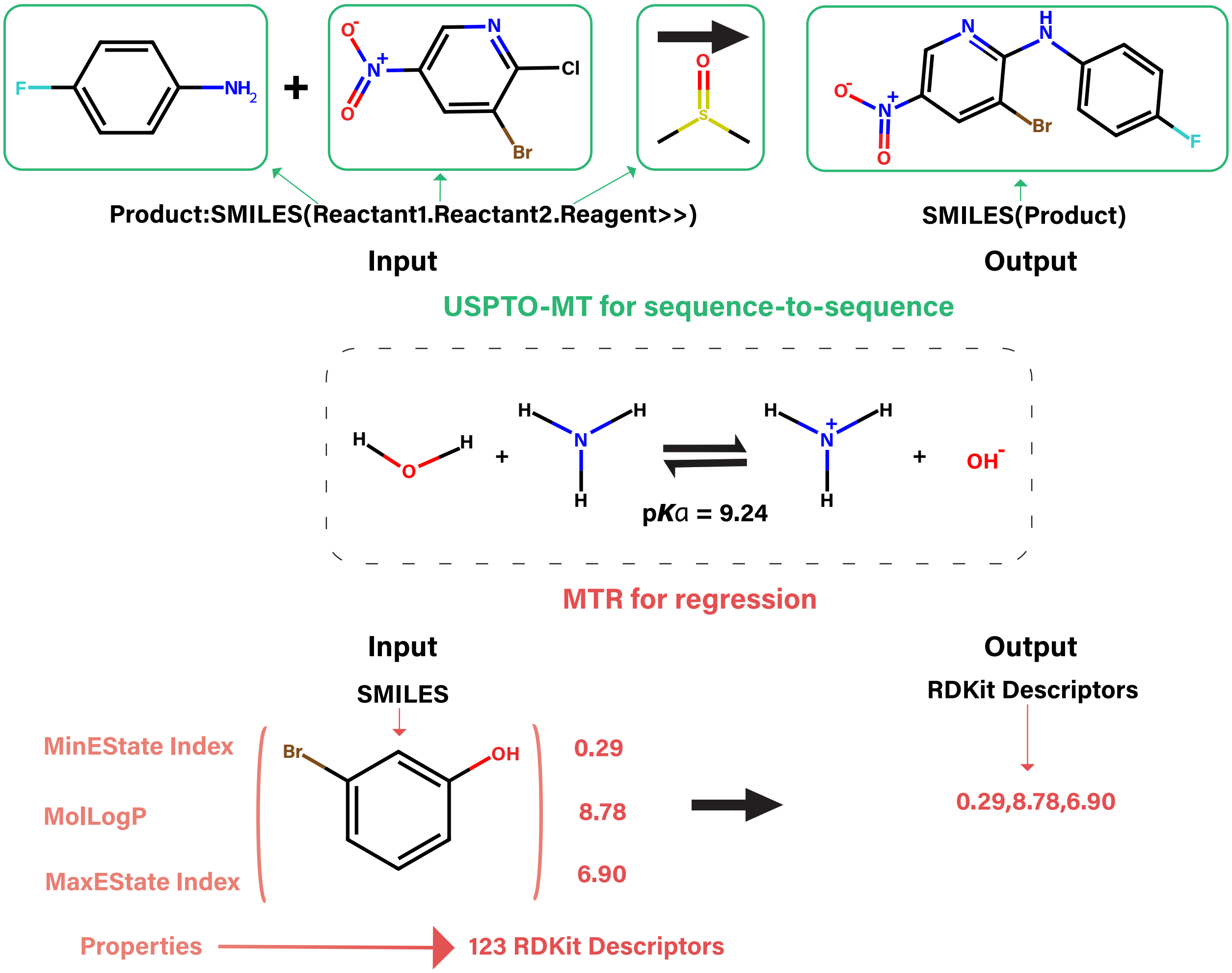

Figure S5: Visualization of the USPTO_500_MT model (in green) and Multi-Target Regression (MTR) (in red) objectives used for pretraining our models. We used transfer learning while training on the calculated pK_a dataset. The USPTO_500_MT pretraining strategy uses a reaction prediction model from the original T5Chem paper. The tasks in this model include: product, reactant, and reagents prediction objectives. The USPTO_500_MT takes a SMILES representation of a reaction that can contain reactants and reagents, and it outputs the product if one of the prefixes was set to "Product". The regression models trained on calculated pK_a values use the MTR pretraining strategy. MTR model was trained to predict the 123 RDKit descriptors for 98 million Pubchem molecules. In the dotted box, we demonstrate how the dissociation of a proton from a molecule can also be translated into a reaction, and with this scheme we can transpose the concept of these pretraining strategies into our own training methods.

Table S2: We use different pretraining strategies, like MTR (multi-target regression) and pK_a values calculated based on the Hammett-Taft Equation used by Schrodinger's Epik, to train our models. When training only on pKaCHU from scratch the results from the ensemble model is significantly worse. Additionally, solely by adding MTR pretrained model into the workflow, the results improve. The final model consists of the pipeline we detail in the paper: training a calculated pK_a model using a MTR pretrained model, then finetuning on the experimental pK_a dataset (pKaCHU).

	**Data Size**	**Scratch (↓)**	**MTR+pKaCHU (↓)**	**Final Model (↓)**
**Novartis**	280	2.21	1.27	0.967
**Literature**	123	1.87	0.699	0.513
**SAMPL6**	10	1.31	1.17	0.865
**SAMPL7**	20	2.41	1.63	0.805
**SAMPL8**	25	2.54	1.19	0.842

Table S3: Top-1 sequence-to-sequence performance across external datasets for models trained using different strategies. Training from scratch resulted in lower accuracy (Acc) and higher rates of invalid (Inv) molecular predictions compared with the final model, which incorporates chemical and ionization information. Initializing from the USPTO_500_MT pretrained model and finetuning on the experimental dataset improved performance relative to training from scratch. Ultimately, the training strategy as described in the paper produced the strongest overall results.

Dataset	**Scratch**		**USPTO+pKaCHU**		**Final Model**	
	Acc (%)	Inv (%)	Acc (%)	Inv (%)	Acc (%)	Inv (%)
**Novartis**	18.9	57.86	89.6	0.71	93.6	0.00
**Literature**	40.7	23.58	99.2	0.00	100.0	0.00
**SAMPL6**	10.0	80.0	90.0	0.00	100.0	0.00
**SAMPL7**	40.0	40.0	100.0	0.00	100.0	0.00
**SAMPL8**	40.0	44.0	88.0	0.00	96.0	0.00

Model Performance and Ensemble Analysis

MicropK_a Model: Ensemble Performance

The performance of each individual model making up the ensemble micropK_a model is shown in Table S4 for the T5pKa models. Using the mean absolute error, we can see that all the models had good variability in Figure S7. Table S5 shows the different RMSE received from

the T5 models split either randomly or by scaffold.

Table S4: Internal test performance of individual T5pKa ensemble members.

Model	MAE	RMSE	R^2	r
1 *random split*	0.533	0.907	0.875	0.939
2 *random split*	0.491	0.788	0.906	0.953
3 *random split*	0.515	0.860	0.888	0.944
4 *random split*	0.554	0.959	0.861	0.931
5 *random split*	0.544	0.933	0.868	0.934
6 *scaffold split*	0.540	0.868	0.886	0.943
7 *scaffold split*	0.563	0.931	0.868	0.933
8 *scaffold split*	0.567	0.899	0.877	0.939
9 *scaffold split*	0.544	0.930	0.869	0.935
10 *scaffold split*	0.546	0.857	0.889	0.945
Ensemble	0.438	0.757	0.913	0.956

Table S5: The microscopic pK_a ensemble comprises five models trained with random splits and five trained with scaffold-based splits. The root-mean-square error (RMSE) for each dataset is reported for the T5-based model.

	Data Size	T5 Random Split (↓)	T5 Scaffold Split (↓)
**Novartis**	280	0.960	1.00
**Literature**	123	0.521	0.520
**SAMPL6**	10	0.823	0.909
**SAMPL7**	20	0.873	0.768
**SAMPL8**	25	0.832	0.865

Microstate Model: sequence-to-sequence

Table S6 shows the training, validation, and internal test results for the sequence-to-sequence model. The assessment of the validity of SMILES is based on RDKit parsing. When a SMILES is considered invalid, it is due to failed canonicalization and/or improper valence assignments. We also notice a direct relationship between Top-K and the invalidity of SMILES, as a beam search in lower probability tokens allows for more structural diversity, but it increases the likelihood for invalid molecules.

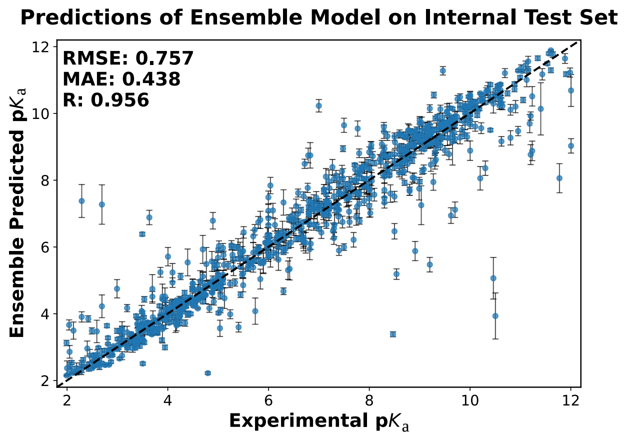

Figure S6: Performance of the microscopic pK_a ensemble model compared with target values. Each point represents the mean prediction across ensemble members, and the vertical error bars indicate the standard deviation of each prediction.

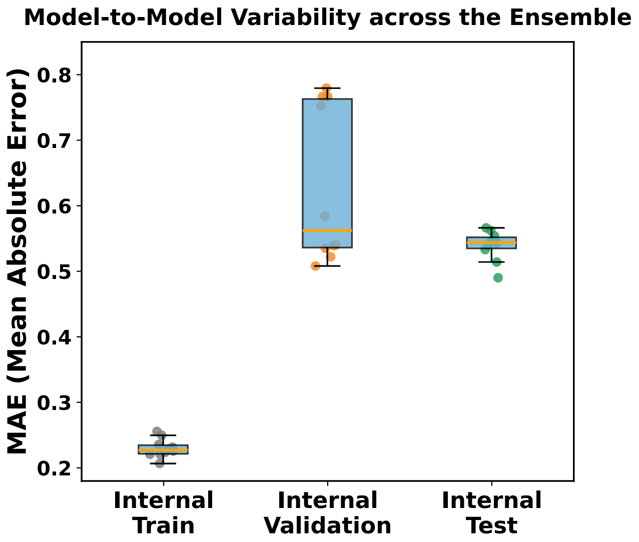

Figure S7: The distribution of mean absolute error (MAE) values across the ten independently trained T5pKa models that make up the ensemble for internal training, internal validation, and internal test sets. The blue boxes represent the inter-quartile range, and the medians are shown as orange horizontal lines. The grey dots denote the individual models in the ensemble for the internal train, the red dots for the internal validation, and the green dots for the internal test.

Table S6: Top-k accuracy and fraction of invalid predictions across training, validation, and test splits.

**Split**	**Top-k**	**Accuracy (%)**	**Invalid (%)**
		**Value**	**Value**
Train	Top-1	100.0	0.00
	Top-2	100.0	15.79
	Top-3	100.0	24.39
	Top-4	100.0	30.15
	Top-5	100.0	34.44
Validation	Top-1	95.6	0.00
	Top-2	98.9	16.94
	Top-3	99.4	25.43
	Top-4	99.4	30.84
	Top-5	99.6	35.42
Test	Top-1	95.6	0.22
	Top-2	99.0	13.03
	Top-3	99.2	21.59
	Top-4	99.3	27.85
	Top-5	99.3	32.55

Polyprotic Molecule Analysis

To analyze the ability of our models on polyprotic molecules, we evaluate the polyprotic molecules present in the internal test set and conduct a focused study on three triprotic amino acids withheld from training: aspartic acid, histidine, and tyrosine. In general, the regression ensemble model demonstrates good generalization to polyprotic molecules. Performance is stronger for diprotic systems compared to the triprotic cases, which are the three amino acids listed, and highlighted by the results for tyrosine in Figure S9. Across all polyprotic molecules in the internal test set, the ensemble model achieves an RMSE of 1.04 and an MAE of 0.56 (Figure S8). The higher sensitivity of RMSE to outliers reflects larger errors observed for certain higher-order ionization steps, like that of tyrosine.

The sequence-to-sequence model also performs well in identifying ionization sites, achieving a Top-1 accuracy of 92% across polyprotic molecules as shown in Table S7. In the triprotic amino acid analysis, the model correctly predicts the ionization site for all but two steps.

Specifically, it predicts the wrong nitrogen on the imidazole ring on histidine on the second ionization. In general, the model struggles with histidine due to the lack of zwitterions available for training.

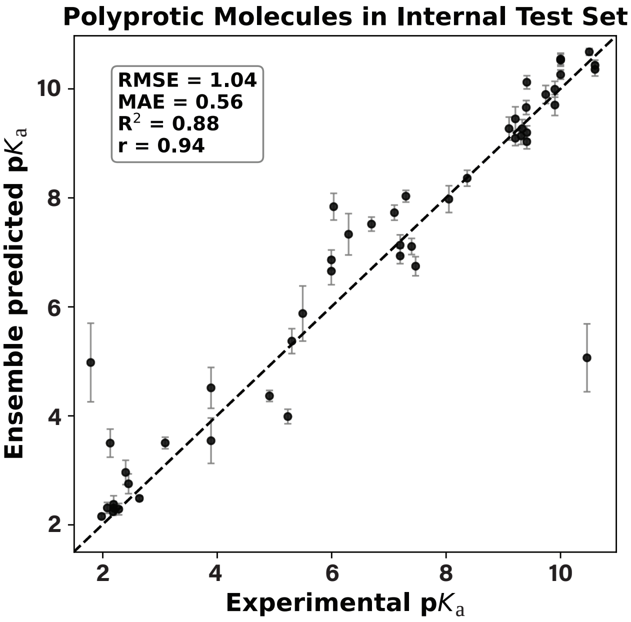

Figure S8: Regression performance of the ensemble model for pK_a prediction on polyprotic molecules in the internal test set (n=50).

Table S7: Sequence-to-sequence microstate prediction performance for polyprotic molecules in the internal test set.

Top-k	Accuracy (%)	Invalid (%)
1	92.0	0.00
2	94.0	18.00
3	94.0	20.67
4	94.0	26.21
5	94.0	27.20

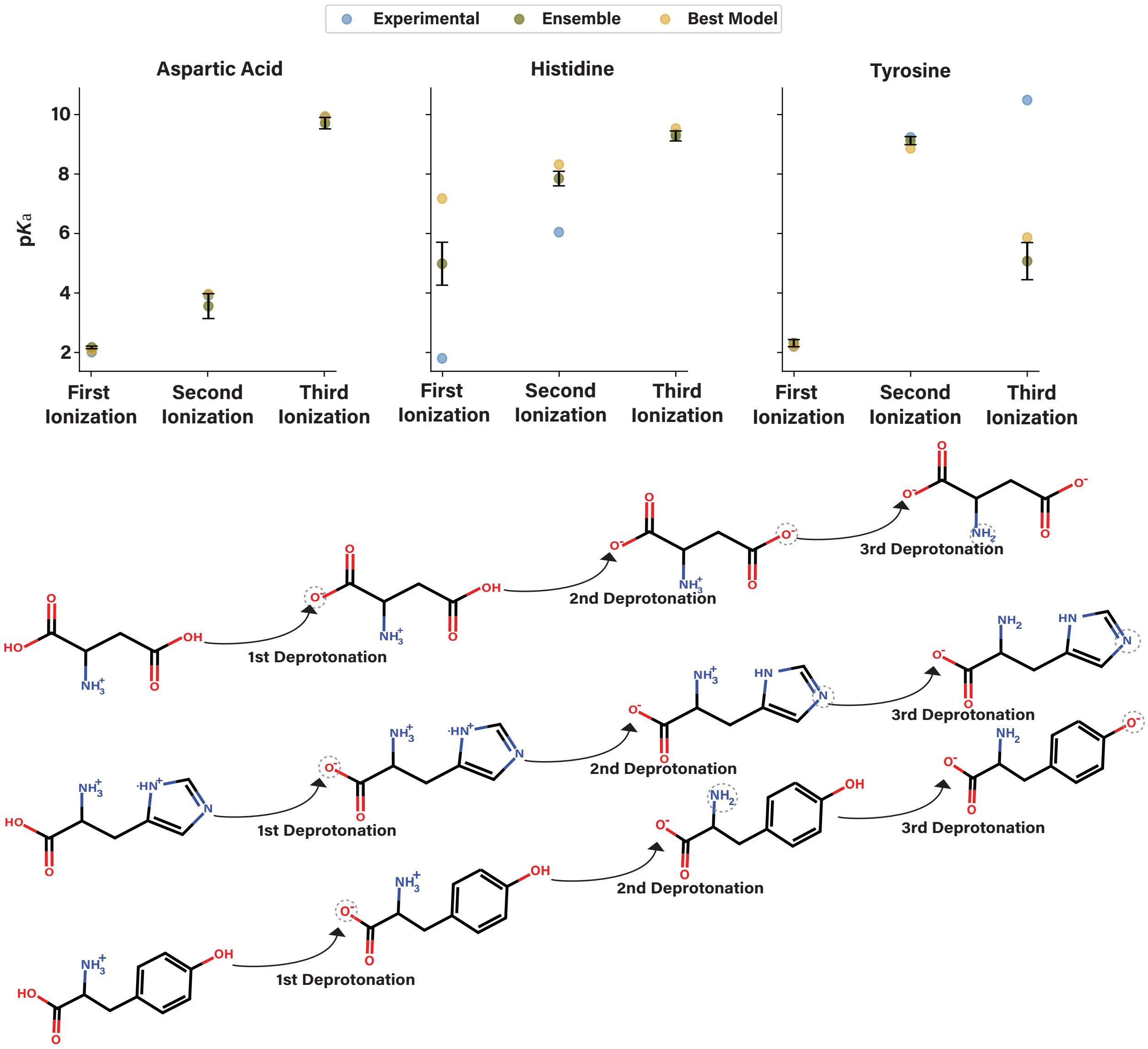

Figure S9: Evaluation of triprotic amino acids not included in the training set. Scatter plots compare experimental values with predictions from both the ensemble model and the selected best model (based on internal test set performance). The sequential deprotonation steps for each molecule are illustrated. The experimentally ionized atom at each step is indicated, while the atom predicted by the sequence-to-sequence model to undergo (de)protonation is highlighted with a gray dashed circle. The sequence-to-sequence model correctly identifies the ionization site for all steps except the second ionization of histidine.

SHAP Analysis

We examined the effect of alcohol substituents and fluorine atoms on the predicted pK_a of pyrrolizidine-based scaffolds (Figure S10) using SHAP values.[5] For the SHAP analysis, SHAP values were computed using the individual ensemble member with the best validation performance, rather than the full ensemble. First, we compare a pyrrolizidine alkaloid with a modified analogue that replaces its secondary alcohol with a methyl group and its primary alcohol is removed. This SHAP analysis shows that replacement of the hydroxyl substituents with a methyl group increases the overall positive contribution to the prediction. This correlates with the higher predicted and experimental pK_a values of the molecule containing the methyl group. This is evident in both the protonated and deprotonated forms, as the region containing the methyl remains positive in both. This is chemically reasonable, since alkyl groups are weak electron-donating groups and tend to increase pK_a. In contrast, the hydroxyl groups present on the molecule contribute negatively to the prediction, lowering the predicted pK_a. This suggests that the model captures chemically meaningful trends, as the negative contributions are associated with the alcohol groups and the ionization region.

In addition, we extended this comparison to fluorinated analogues of the pyrrolizidine scaffold. In these structures, replacement of the alcohol-containing substituents with fluorine atoms led to a further decrease in the predicted pK_a. Consistent with the strong electron-withdrawing character of fluorine, the SHAP values show a decrease from the C=C bond and a more negative contribution near the ionizable atom. Therefore, this study follows the patterns seen in experiments as methyl substitution increases basicity and hydroxyl and/or fluorination increases acidity.[6]

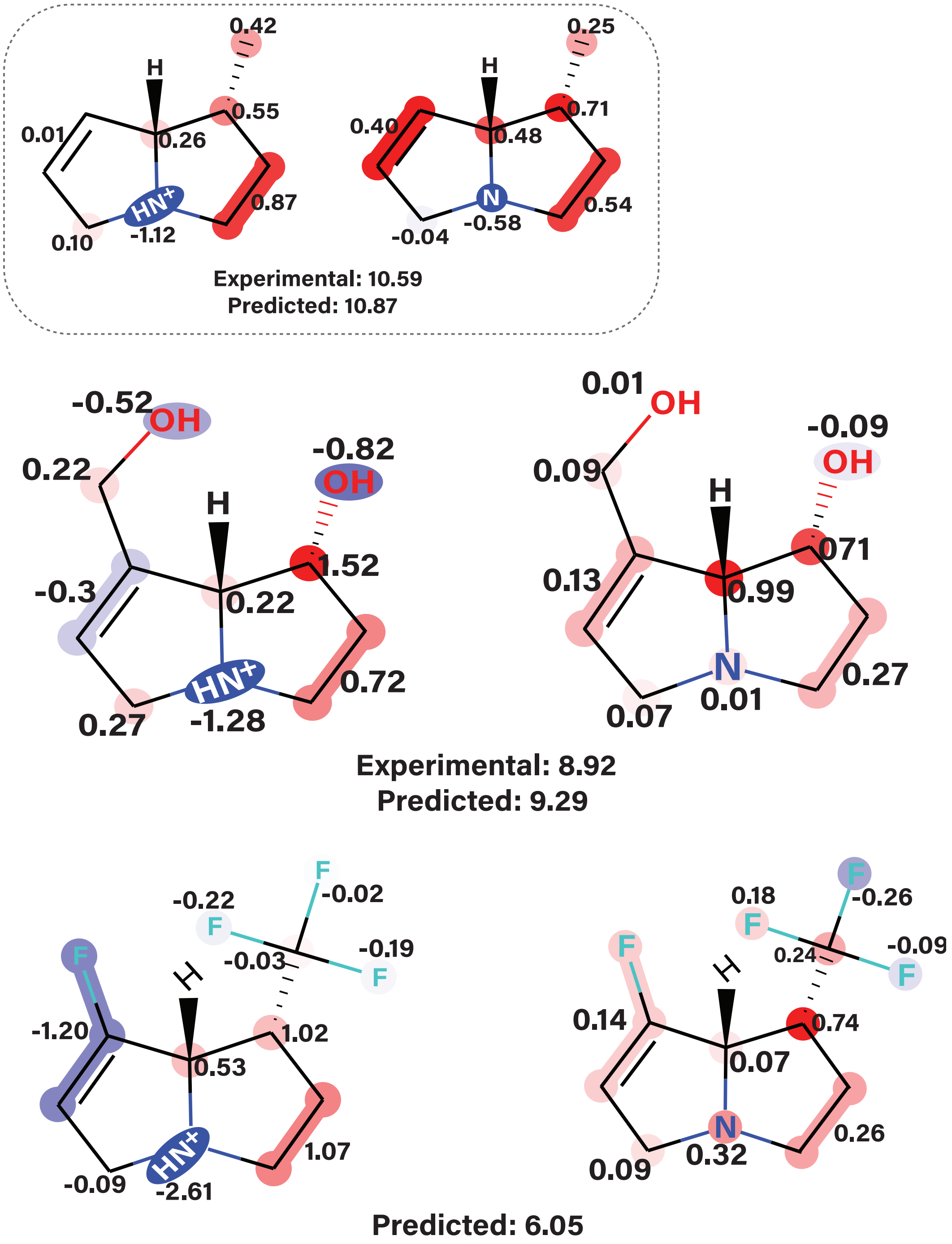

Figure S10: SHAP analysis of pyrrolizidine-based scaffolds illustrating the effect of alcohol substituents and fluorine atoms on the predicted pK_a. The pyrrolizidine alkaloid is compared with a modified analogue, which replaces its secondary alcohol group with a methyl group. The primary alcohol group is removed. Removal of the hydroxyl groups increases the positive contribution (red) to the prediction. While the hydroxyl groups contribute negatively (blue) to the overall pK_a. In a further modification, the alcohol groups were replaced with fluorine atoms to increase acidity. As expected, the predicted pK_a decreased upon fluorination.

SAMPL6 NMR molecules

SAMPL6[7] reports NMR data for two molecules that includes their ionization site and pK_a value: SM07 and SM14. Based on structural similarity, five additional 4-amino quinazoline derivatives were derived from SM07 and SM14. Their corresponding spectroscopic pK_a values were inferred as microscopic pK_a values due to their parent compounds. In this study, the SAMPL6 dataset was obtained from the official SAMPL6 repository, which accompanies the published benchmark.

In Figure S11, we illustrate the alternative tautomeric forms for each 4-amino quinazoline molecule in the SAMPL6 NMR dataset. To determine the relative stability of these tautomers, we evaluated each neutral molecule using sPhysNet-Taut[8] model for tautomer stability prediction. Since sPhysNet-Taut cannot predict charged tautomers, only SM07 and its derivatives were considered.

Based on the results from sPhysNet-Taut (Table S8), T5pKa is able to predict the correct ionization region, when it is provided with the most stable neutral tautomer as input. Additionally, we evaluated the ensemble micropK_a regression model using either the stable or less stable tautomer as input. The model receiving the more stable tautomeric input resulted in an RMSE of 0.865, and the less stable tautomeric inputs resulted in an RMSE of 0.868. This indicates that the model's performance is not sensitive to tautomer stability.

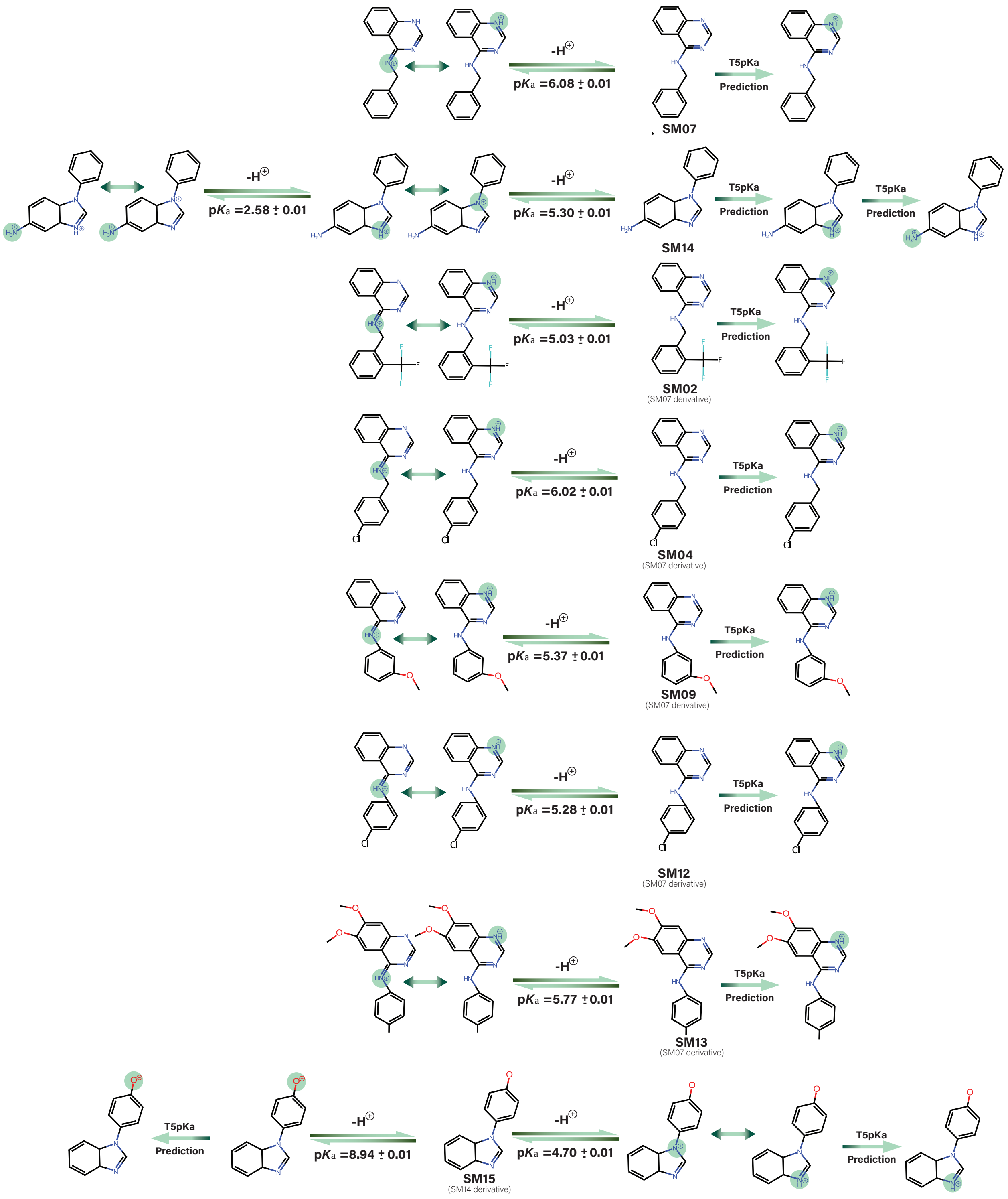

Figure S11: Illustration of SAMPL6 molecules. The prediction and targets are tautomers of each other.

Table S8: SM07 from the SAMPL6 NMR study exists in two tautomeric forms. Consequently, we used sPhysNet-Taut to identify the more stable tautomer and applied the same procedure to all SM07 derivatives. In accordance with the original sPhysNet-Taut protocol, low-energy tautomeric states were identified using an energy cutoff of 2.76 kcal/mol. Molecule IDs follow the SAMPL6 documentation, and each tautomer is represented by the predicted energy ranking (kcal/mol), stability assignment, and corresponding pK_a value.

**Molecule ID**	**SMILES**	**Score (kcal/mol)**	**Label**	**pK_a**
SM07	c1ccc(CNc2ncnc3ccccc23)cc1	0.00	low energy	6.08
SM07	c1ccc(CN=c2nc[nH]c3ccccc23)cc1	4.48	high energy	6.08
SM03 (SM07 derivative)	FC(F)(F)c1cccc(Nc2ncnc3ccccc23)c1	0.00	low energy	5.03
SM03 (SM07 derivative)	FC(F)(F)c1cccc(N=c2nc[nH]c3ccccc23)c1	3.00	high energy	5.03
SM04 (SM07 derivative)	Clc1ccc(CNc2ncnc3ccccc23)cc1	0.00	low energy	6.02
SM04 (SM07 derivative)	Clc1ccc(CN=c2nc[nH]c3ccccc23)cc1	4.04	high energy	6.02
SM09 (SM07 derivative)	COc1cccc(Nc2ncnc3ccccc23)c1	0.00	low energy	5.37
SM09 (SM07 derivative)	COc1cccc(N=c2nc[nH]c3ccccc23)c1	2.43	low energy	5.37
SM12 (SM07 derivative)	Clc1cccc(Nc2ncnc3ccccc23)c1	0.00	low energy	5.28
SM12 (SM07 derivative)	Clc1cccc(N=c2nc[nH]c3ccccc23)c1	2.42	low energy	5.28
SM13 (SM07 derivative)	COc1cc2ncnc(Nc3cccc(C)c3)c2cc1OC	0.00	low energy	5.77
SM13 (SM07 derivative)	COc1cc2[nH]cnc(=Nc3cccc(C)c3)c2cc1OC	3.87	high energy	5.77

References

(1) Lu, J.; Zhang, Y. Unified Deep Learning Model for Multitask Reaction Predictions with Explanation. *Journal of Chemical Information and Modeling* **2022**, *62*, 1376–1387, PMID: 35266390.

(2) Gaulton, A.; Bellis, L. J.; Bento, A. P.; Chambers, J.; Davies, M.; Hersey, A.; Light, Y.; McGlinchey, S.; Michalovich, D.; Al-Lazikani, B.; Overington, J. P. ChEMBL: a large-scale bioactivity database for drug discovery. *Nucleic Acids Research* **2011**, *40*, D1100–D1107, PMID: 21948594.

(3) Kim, S.; Chen, J.; Cheng, T.; Gindulyte, A.; He, J.; He, S.; Li, Q.; Shoemaker, B. A.; Thiessen, P. A.; Yu, B.; Zaslavsky, L.; Zhang, J.; Bolton, E. E. PubChem 2023 update. *Nucleic Acids Research* **2022**, *51*, D1373–D1380.

(4) RDKit: Open-Source Cheminformatics Software, version 2022.03.2. `https://www.rdkit.org`.

(5) Lundberg, S. M.; Lee, S.-I. A Unified Approach to Interpreting Model Predictions. Advances in Neural Information Processing Systems. 2017.

(6) An, H.; Liu, X.; Cai, W.; Shao, X. Explainable Graph Neural Networks with Data Augmentation for Predicting pKa of C–H Acids. *Journal of Chemical Information and Modeling* **2024**, *64*, 2383–2392, PMID: 37706462.

(7) Işık, M.; Rustenburg, A. S.; Rizzi, A.; Gunner, M. R.; Mobley, D. L.; Chodera, J. D. Overview of the SAMPL6 pKa Challenge: Evaluating small molecule microscopic and macroscopic pKa predictions. *Journal of computer-aided molecular design* **2021**, *35*, 131–166.

(8) Pan, X.; Zhang, X.; Xia, S.; Zhang, Y. Fast and Accurate Prediction of Tautomer Ratios in Aqueous Solution via a Siamese Neural Network. *Journal of Chemical Theory and Computation* **2025**, *21*, 3132–3141.
